# The uncertain representation ranking framework for concept-based video retrieval

**DOI:** 10.1007/s10791-012-9207-y

**Published:** 2012-07-21

**Authors:** Robin Aly, Aiden Doherty, Djoerd Hiemstra, Franciska de Jong, Alan F. Smeaton

**Affiliations:** 1 Database Group and Human Media Interaction Group, University of Twente, Enschede, The Netherlands; 2 British Heart Foundation Health Promotion Research Group, University of Oxford, Oxford, UK; 3 CLARITY: Centre for Sensor Web Technologies, Dublin City University, Dublin, Ireland

**Keywords:** Representation uncertainty, Concept-based representation, Video retrieval

## Abstract

Concept based video retrieval often relies on imperfect and uncertain concept detectors. We propose a general ranking framework to define effective and robust ranking functions, through explicitly addressing detector uncertainty. It can cope with multiple concept-based representations per video segment and it allows the re-use of effective text retrieval functions which are defined on similar representations. The final ranking status value is a weighted combination of two components: the expected score of the possible scores, which represents the risk-neutral choice, and the scores’ standard deviation, which represents the risk or opportunity that the score for the actual representation is higher. The framework consistently improves the search performance in the shot retrieval task and the segment retrieval task over several baselines in five TRECVid collections and two collections which use simulated detectors of varying performance.

## Introduction

Concept-based video retrieval has many advantages over other content-based approaches (Snoek and Worring 2009). In particular, it is more straightforward to define ranking functions on concept-based representations than for most other content-based representations (Naphade et al. 2006). For example, the definition of a ranking function for the query “Find me tigers” is intuitively more straightforward based on the concept *Animal* in a (video-) segment 1 than based on the color distribution in an example image. As the current state-of-the art in automatic concept detection is not mature enough for ranking functions directly using the binary concept labels occurs/absent (Hauptmann et al. 2007), concept-based search engines use the confidence score of a detector that the concept occurs. However, the uncertainty introduced by the use of confidence scores makes the definition of effective and robust ranking functions again more difficult. This paper presents a general framework for the definition of concept-based ranking functions for video retrieval that fulfill these requirements.

Research in concept-based retrieval currently focuses on the retrieval of video shots, which are segments of roughly five seconds length. According to Kennedy et al. (2008) the main problem here is the definition of query-specific ranking functions, which are often modeled as weighted sums of confidence scores. But the estimation of weights based on semantic distance of the concept to the query or on relevance feedback has proven difficult, which leads to poor performance (Aly et al. 2009). Another approach learns weights for a set of query classes based on relevance judgments for training queries (Yan 2006). However, the gathering of relevance judgments for training queries is expensive and it is unclear how to define a suitable set of query classes. Additionally, although de Vries et al. (2004) find that users do not only search for shots but also for longer segments, concept-based search engines do not support this retrieval task. A likely reason is that a single confidence score per segment does not sufficiently discriminate relevant from nonrelevant segments. However, it is not straightforward to define a more discriminative document representation based on confidence scores. Therefore it is an important challenge to come up with a framework to define ranking functions for varying retrieval tasks that are *effective* for arbitrary queries.

The performance of detectors changes significantly with the employed detection technique and the considered collection (Yang and Hauptmann 2008). If a ranking function strongly depends on a particular distribution of confidence scores, its performance varies, which is clearly undesirable. For example, the confidence scores of the concept *Animal* in relevant shots for the query “Find me tigers” can be high in one collection and low in another collection. Now, if a ranking function assumes that confidence scores for the concept *Animal* in relevant shots are high, its performance will be poor for the second collection. Because current ranking functions are weighted sums of confidence scores they rely on the weight estimation to adapt the weights according to the score distribution of the considered collection. However, how could we estimate these weighted for arbitrary detectors and collections? Therefore it is also an important challenge to define *robust* ranking functions over detectors of varying performance.

In this paper, we propose the uncertain representation ranking (URR) framework which describes a general way to define ranking functions which meet the following challenges:

- they are *effective* for arbitrary queries, and
- they are *robust* over detector techniques and collections.

The framework uses a basic ranking function defined on representations of binary concept labels and addresses the uncertainty of the concept detectors separately. In this paper, we adapt *effective* ranking functions from text retrieval. To address detector uncertainty, the framework considers multiple representations for each document. Applying the basic ranking function to each representation leads to multiple possible retrieval scores for each document. The final score is a combination of the expected score, which represents a good guess of the score of a known representation, and the scores’ standard deviation, which represents the chance that the score is actually higher or lower. Taking into account the expected score makes the performance *robust* against changes of detectors and collections. This paper focuses on the definition of concept-based ranking functions. For this purpose we use results of existing work for the setting of the ranking functions’ parameters.

To demonstrate that the framework produces effective and robust ranking functions, we show that this is the case for the shot retrieval task and the segment retrieval task. Note that the ranking functions used for these tasks originate from ideas which we proposed earlier. In Aly et al. (2008) we propose to rank shots by the probability of relevance given the confidence scores, marginalizing over all possible concept occurrence. The ranking function obtained through marginalization is equal to the expected score used in the URR framework. The expected score allows us to additionally model the risk of choosing a certain score. Furthermore, in Aly et al. (2010) we propose a ranking function for segment retrieval, where the idea of ranking by the expected score and the scores’ standard deviation is used for the first time for a concept language model ranking function and a document representation in terms of concept frequencies. The URR framework generalizes this idea to arbitrary ranking functions and representations.

The remainder of this paper is structured as follows. First in Sect. 2 related work on treating uncertainty in information retrieval is presented. In Sect. 3 we describe the proposed URR framework. In Sects. 4 and 5 the framework is applied to shot and segment retrieval respectively. Then Sect. 6 describes the experiments which we undertook to evaluate the URR framework. Section 7 discusses the experimental results. Finally, Sect. 8 presents the conclusions.

## Related work

In this section we describe how related work approaches uncertainty, both in concept-based video retrieval and in text retrieval. Note that there are significant bodies of research on the storage of uncertainties in databases, see for example Benjelloun et al. (2006), and on the exploitation of uncertain knowledge representations for the inference of new knowledge, see for example Ding and Peng (2004), which lie outside the scope of this paper.

### Concept-based video ranking functions

Most concept-based video ranking functions use confidence scores of detectors built from support vector machines. To ensure comparability of confidence scores among concepts, confidence scores are usually normalized. Platt (2000) provides a method to transform a confidence score into a posterior probability of concept occurrence given the confidence score, which we refer to as probabilistic detector output.

**Fig. 1:**
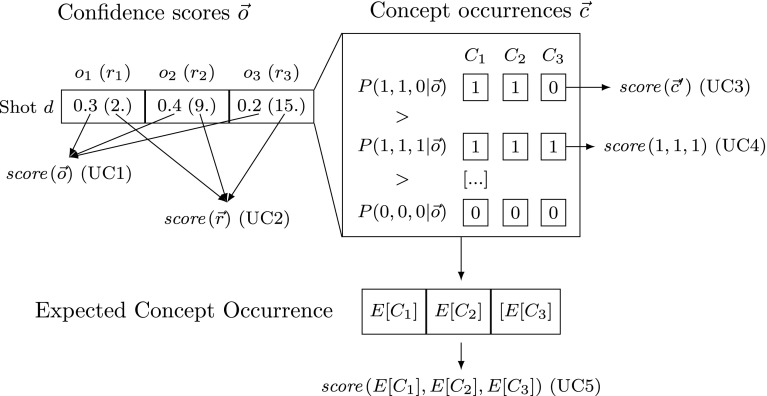
Uncertainty classes (UC1–UC5) of video shot ranking functions *score* using three concepts: confidence score-based (**o**) (UC1), on the rank of confidence scores (**r**) based (UC2), based on the most likely concept representation (**c**′) (UC3), based on the probability that all concepts occur (UC4), and based on the expected concept occurrences (UC5)

Figure 1 shows a classification of existing concept-based ranking functions into principle ways of dealing with detector uncertainty, to which we refer to as uncertainty classes. On the left the figure shows the confidence scores **o** for the three concepts of a shot together with their ranks within the collection. The confidence scores are then used to determine the posterior probability of each possible concept representations. At the bottom the occurrence probabilities for each concept are combined into the expected concept occurrence. In the following we will describe well-known methods of each uncertainty class.

In uncertainty class UC1, ranking functions (indicated by *score*) take confidence scores as arguments. Most ranking functions are weighted sums or products of confidence scores, where the used weights carry no particular interpretation (Snoek and Worring 2009). Yan (2006) proposes the Probabilistic Model for combining diverse knowledge sources in multimedia. The proposed ranking function is a discriminative logistic regression model, calculating the posterior probability of relevance given the observation of the confidence scores. Here the confidence score weights are the coefficients of the logistic regression model. The ranking functions of uncertainty class UC1 mainly have the problem that they require knowledge about the confidence score distributions in relevant shots, which is difficult to infer. Additionally, if a concept detector changes, the distribution of confidence scores changes, making existing knowledge obsolete.

In uncertainty class UC2, ranking functions are based on the (inverse) rank of the confidence scores within the collection (McDonald and Smeaton 2005; Snoek et al. 2007). As only the ranks of confidence scores are taken into account, estimating weights for this uncertainty class only requires knowledge over the distribution of confidence scores in relevant shots relative to other shots. Otherwise UC2 suffers from the same drawbacks as UC1.

In uncertainty class UC3, ranking functions take a vector of the most probable concept representation as arguments. To the best of our knowledge, no method of this class was proposed in concept-based video retrieval so far, most likely due to the weak performance of concept detectors. Nevertheless, we include this uncertainty class in our discussion because methods of this class have been used in spoken document retrieval, where the most probable spoken sentence is considered (Voorhees and Harman 2000), and once concept detectors improve, ranking functions from this class might become viable.

In uncertainty class UC4, ranking functions use a particular concept representation, not necessarily the most probable, together with its probability. Zheng et al. (2006) propose the point-wise mutual information weight (PMIWS) ranking function. As we showed in Aly et al. (2008), the PMIWS can be seen to rank by the probability of relevance given the occurrence of all selected concepts multiplied by the probability that these concepts occur in the current shot. The main problem of instances of uncertainty class UC4 is that concepts which only occur sometimes in relevant shots cannot be considered. To see this, let us assume perfect detection, a concept that occurs in 50 % of the relevant shots, and a ranking function that only rewards shots in which this concept occurs. Here, relevant shots, in which the concept does not occur, receive zero score.

In uncertainty class UC5, ranking functions take the expected components of concept occurrences as parameters. Li et al. (2007) propose an adaptation of the language modeling framework (Hiemstra 2001) to concept-based shot retrieval. We show in (Aly 2010, p. 32) that the ranking function by Li et al. (2007) can also be interpreted as using the expected concept occurrence in the language modeling framework where concepts (terms) either appear or not. Instead of focusing on one representation, as done by UC3 and UC4, this uncertainty class combines all possible representations into the expected values of a representation, which is then used in a ranking function. The ranking functions of uncertainty class UC5 are limited to arguments of real numbers because they are defined on expectations, which are real numbers. But some existing effective probabilistic ranking functions, for example the binary independence model (Robertson et al. 1981), are defined on binary arguments, and therefore cannot be used. Furthermore, the ranking functions in uncertainty class UC5 result in a single score, which abstract from the uncertainty that is involved by using this result.

The URR framework proposed in this paper can be seen as a general ranking framework of a new uncertainty class (UC6) of ranking functions that are defined on the distribution of all possible concept-based representations of a document. The URR framework uses a basic ranking function to calculate a score for each possible representation. The final ranking score value of a document is then calculated by combining the expected score and the scores’ standard deviation according to the probability distribution over the possible representations for this document. This procedure has the following advantages. Compared to the uncertainty classes UC1 and UC2, the basic ranking function of the URR framework does not require knowledge about the distribution of confidence scores in relevant segments. In contrast to the uncertainty classes UC3 and UC4, which both only use a single concept-based representation, the URR framework takes into account all possible representations, which reduces the risk of missing the actual representation of a document. Finally, compared to uncertainty class UC5, the basic ranking functions in the URR framework are defined on concept-based representations, which allow us to re-use existing, effective ranking functions from text retrieval. Additionally, the scores’ standard deviation in the URR framework can be seen as a measure of the riskiness of score, which we show can be used in ranking.

### Uncertainty in text retrieval

We are not the first to address uncertainty in information retrieval, which has been done before in text retrieval, for example, in probabilistic indexing and in the recently proposed mean-variance analysis framework for uncertain scores, as well as in several other areas. We describe the former two approaches in the following.

#### Probabilistic indexing

In probabilistic indexing for text retrieval, the assignment of an index terms to a document is only probabilistically known. Croft (1981) approaches this uncertainty by ranking documents according to the expected score of the binary independence ranking function (Robertson et al. 1981). However, Fuhr (1989) shows that, although the binary independence ranking function is a rank preserving simplification of the probability of relevance function, the expected binary independence score is not rank preserving to the expected probability of relevance score. Instead, Fuhr (1989) ranks by the probability of relevance given the confidences of indexers as a ranking function, marginalizing over all possible index term assignments. This marginalization is equivalent to ranking by the expected probability of relevance, which we use as a ranking component of our URR framework in Sect. 4.

Note that there is a difference in interpretation between the marginalization and the expected score used in the URR framework, which we discuss in the following. The marginalization approach considers for each document the probability of relevance of *any* document with the same indexer confidences, which are similar to confidence scores in concept-based video retrieval. On the other hand, the URR framework uses the expected score of a *particular* document. This allows us to consider the scores’ standard deviation, which represents the risk or opportunities of ranking a document by its expected score. Additionally, Fuhr assumes that the true index term assignments of a document are always unknown, but for the URR framework concept occurrences are only uncertain because of the uncertainty of detectors. Indeed, the URR framework could be extended to handle the case where the occurrences of some concepts are known, which we propose for future work. Additionally to the expected score, the URR framework considers a component to represent the risk inherent to a retrieval model when ranking a document.

#### Mean-variance analysis

Wang (2009) proposes the mean-variance analysis framework for managing uncertainty in text retrieval, which is based on the Portfolio Selection Theory (Markowitz 1952) in finance. We believe that the processes in finance are more intuitive, therefore we first describe the Portfolio Selection Theory and describe its application to text retrieval afterwards.

The Portfolio Selection Theory finds efficient portfolios based on the uncertain future win of companies in a portfolio. The win of a portfolio is:

1 $$ Win=\sum_{j=1}^N{p_j\;d_j}. Win $$

where *Win* is the random variable of the total win of the portfolio, *d*_*j*_.*Win* > 0 is the random variable of company *d*_*j*_’s win 2, and *p*_*j*_ (with 0 ≤ *p*_*j*_ ≤ 1 and ∑_*j*_ *p*_*j*_ = 1) is the percentage of the available budget invested in company *d*_*j*_. The Portfolio Selection Theory assumes that analysts can predict the following statistical components for a company *d*_*j*_:

1. The *expected* win, *E*[*d*_*j*_.*Win*] (“What win is to be expected from the company *d*?”).
2. The *variance* of the win, *var*[*d*_*j*_.*Win*] (“How widely do the possible wins vary?”).
3. The *co-variance* between the win of company *d* and any other company *d*_*j*_,  cov[*d*_*j*_.*Win*, *d*_*i*_.*Win*] (“How does the win of company *d*_*j*_ influence the win of company *d*_*i*_?”).

**Fig. 2:**
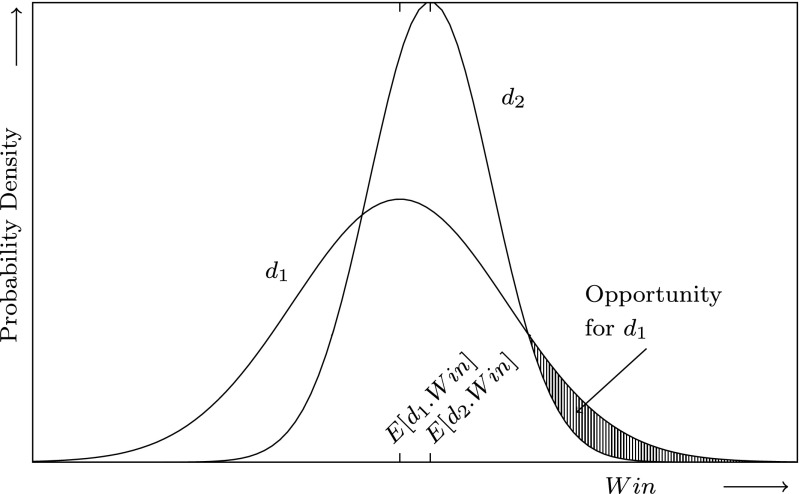
The win distributions of company *d*_1_ and company *d*_2_. The area marked as “Opportunity for *d*_1_” shows the reason why a risk-loving investor (*b* < 0), would buy companies of *d*_1_ (*E*[*d*.*Win*] is the expected win and the variance of the win is implicitly specified by the shape of the Gaussian)

The above statistical components are then used to find an efficient portfolio, a set of percentages $(p_1,\ldots,p_N)$, which optimizes the following expression:

2 $$ E[Win]-b\; \hbox{var}[Win] $$

where *b* is the *risk parameter* which represents the risk-attitude of the analysts. If *b* > 0, analysts are *risk-averse*. For *b* = 0, analysts would only invest in the company of the highest expected win, which Markowitz (1952) identified as unreasonable in finance as the whole budge would be invested in the company with the highest expected win. If *b* < 0, analysts like to take risks, which we informally call *risk-loving*. Figure 2 shows an example of the win distributions of two companies *d*_1_ and *d*_2_ ignoring the co-variance between their wins. Intuitively, *risk-averse* and *risk-neutral* analysts invest everything into company *d*_2_ (*p*_1_ = 0, *p*_2_ = 1) because it has a higher expected win. However, *risk-loving* analysts speculate on a win of company *d*_1_ in the area denoted by “Opportunity for *d*_1_” and therefore will increase *p*_1_.

In the mean-variance analysis framework, a document *d* is equivalent to a company and the uncertain score *d*.*S* of document *d* is equivalent to the uncertain win *d*. *Win* of the company *d*. For a ranking function *s* the mean-variance analysis assumes that the expected score of a document is *E*[*d*.*S*] = *score*(**f**), where **f** is the known representation of document *d*. Wang (2009) transforms the Portfolio Selection criterion from Eq. (2) into a document ranking problem by fixing a percentage *p*_*i*_ to rank *i* rather than to a document and requires that weights monotonically decrease (*p*_*i*_ > *p*_*i*+1_). Therefore, it is no longer a question as to what percentage to invest, but how to rank documents. In contrast to the Portfolio Selection Theory, where a risk-neutral attitude *b* = 0 leads to unwanted results, a risk-neutral attitude is an intuitive solution in the mean-variance analysis framework because the expected value is an unbiased estimator of the actual score (Papoulis 1984). Therefore, the scores’ variance only adds something on top of an already reasonable solution rather than making the solution reasonable, which is the case in the Portfolio Selection Theory. For the transformed Portfolio Selection Theory formula in Eq. (2) to the document ranking problem with fixed percentages, Wang (2009) proposes a greedy algorithm as a solution, which ranks a document *d* ^*^ at rank *j* which has the highest mean-variance trade-off:

3 $$ d^* = \hbox{argmax}_{d} \left( E[d.S]-\; b\; p_j\; \hbox{var}[d.S] -\; 2b \sum_{k=1}^{j-1}{p_j\; p_k\; \hbox{cov}[d.S,d_{k}.S]} \right) $$

where $d_1, \ldots, d_{j-1}$are the previously ranked documents. In an analogy to the Portfolio Selection Theory, the mean-variance analysis requires estimations for the variance and co-variance of the ranking status value, which Wang (2009), Wang and Zhu (2009) provide.

The URR framework uses a similar ranking algorithm to the one proposed in Eq. (3), using the scores’ standard deviation instead of its variance. In the mean-variance analysis, the reason for the uncertainty of a document’s score is unspecified. On the other hand, in the URR framework the scores’ standard deviation originates from the uncertain document representation. Similar to the mean-variance analysis, the URR framework could also take into account correlations between document representations, to influence the standard deviation of the score. For example, videos usually follow a story and the occurrence of concepts in nearby shots are correlated (the fact that an *Animal* occurs in a shot influences the probability of an *Animal* in a nearby shot). Yang and Hauptmann (2006) are the first to explore the exploitation of such correlations in videos. As until now only oracle models trained on the test collection were able to achieve significant improvements, we leave the consideration of co-variances, although promising, to future work.

## The uncertain representation ranking framework

This section describes the URR framework which ranks segments by considering uncertain concept-based representations in a similar way as the Mean-Variance framework (Wang 2009)^3^.

### Intuitive example

**Fig. 3:**
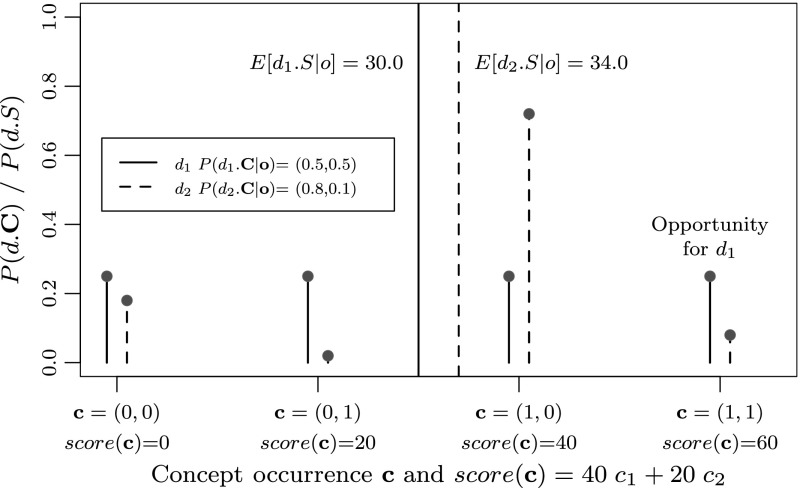
The score distributions for document *d*_1_ and document *d*_2_ considering two concepts. *P*(*d*.**C** = **c**|**o**) is the probability that the actual concept occurrences are **c**

Before we formally define the URR framework we introduce an intuitive example using a particular document representation and ranking function. Let us consider a collection of two documents and *n* = 2 concepts. Furthermore, let us assume that an effective ranking function based on known concept occurrences for the current query would be the following:

4 $$ score({{\bf c}})=\sum_{i=1}^{n} w_i\; c_i $$

where **c** is a binary vector of concept occurrences, *score*(**c**) is the ranking function, *c*_*i*_ is a concept occurrence state of concept *i* (*c*_*i*_ = 1 if it occurs), and *w*_*i*_ is the weight for concept *i*. For this example, let *w*_1_ = 20 and *w*_2_ = 40. We denote the uncertain concept occurrences in document *d* by the random variable *d*.*C*. We assume that concept detectors can predict the occurrence of a concept probabilistically. For example, given a confidence score *o*_*d*,*i*_ for document *d*, the probabilistic output of a concept detector for concept *i*, would be *P*(*d*.*C*_*i*_|*o*_*d*,*i*_). For each document, there are 2^*n*^ possible combinations of *n* concept occurring or being absent, which we jointly denote by a vector of random variables *d*.**C**. The probabilities of the occurrence of each of the *n* concepts given the confidence scores **o** can then be combined to the posterior probability of each combination concept stats **c** (a binary vector), *P*(*d*.**C** = **c**|**o**). According to the ranking function in Eq. (4), each state combination **c** results in a score. We denote the uncertain score of each document *d* as *d*.*S* = *score*(*d*.**C**), a function of random variables, which is again a random variable^4^. From the above, we can calculate the expected score of a document *d*,  *E*[*d*.*S*|**o**], and it’s standard deviation $\sqrt{\hbox{var}[d.S|{\bf o}]}$. Figure 3 visualizes this scenario (the standard deviation is represented by the spread of the distribution).

A search engine in a risk-neutral will rank document *d*_2_ above document *d*_1_ because it has a higher expected score. However, similar to the analysts in the previous section, the search engine in a risk-loving setting might prefer document *d*_1_ over document *d*_2_ because of the higher probability that the document has the highest score of 60. In the following section we define the URR framework, which generalizes this intuitive case to arbitrary score functions defined on arbitrary concept representations.

### Definitions

Because the URR ranking framework is not specific to a particular type of feature, let ${\bf F}=(F_1,\ldots,F_n)$ be the considered representation of documents for the current query consisting of *n* features (or representation). Formally, each feature *F*_*i*_ is a random variable, a function of documents to feature values. For example, the ranking functions in this paper consider concept occurrences, denoted by *C*s, and concept frequencies, denoted by *CF*s, as features. For the query “Find me tigers”, a search engine might consider the frequencies of the concept *Animal* and the concept *Jungle* **CF** = (*CF*_1_,*CF*_2_) as features where *CF*_1_(*d*) and *CF*_2_(*d*) yield the frequency of the concept *Animal* and the concept *Jungle* in document *d* respectively.

Furthermore, let $score: rng({\bf F}) \rightarrow \hbox{IR}$ be a ranking function which maps known feature values to scores, where $rng(\cdot)$ denotes the range of a function. For example, the simple ranking function in Eq. (4), $score({\bf f} \in rng({\bf F}))=\sum\nolimits_i{w_i\; f_i}$ where *w*_*i*_ is the weight feature value *f*_*i*_, is such a score function. Note that we adopt the common notation of random variables and denote random variables and functions in the same way as their range, therefore leaving out $rng(\cdot)$ in the following (Papoulis 1984).

Because the feature values of documents are uncertain, we introduce the random variable *d*.**F** for the feature values of document *d*. Furthermore, let *d*.*S* = *score*(*d*.**F**) be the random variable for the score of document *d* which results from the application of the ranking function *score* on *d*’s uncertain feature values *d*.**F**. For example, if a segment contains *m* shots and the considered representation consists of *n* concept frequencies, the random variable of the uncertain concept frequencies **CF** *d* ranges over (*m* + 1)^*n*^ possible frequency combinations, and the random variable *d*.*S* ranges over the scores obtained from the application of *score* on each combination.

It is important to note the difference between the random variables **F** and the ranking function *score* on the one hand, and its document-specific counter parts *d*.**F** and *d*.*S* on the other hand. For example, *score*(**F**(*d*)) is the actual score of document *d* based on the known features **F**(*d*). On the other hand, *d*.**F** and *d*.*S* are random variables for the possible feature values and their corresponding scores of document *d*.

We denote the posterior probability of a document *d* having representation values $f \in d.{\bf F}$ given the confidence scores **o** as *P*(*d*.**F** = **f**|**o**), which we use to calculate the expected score and its standard deviation.

### Ranking framework

Using the above definitions we now define statistical components of the URR framework, the expected score and the scores’ variance. The most important component of the URR framework is the expected score of a document *d*. That is, if we consider the representation of *d* to be random, what score do we expect on average. As the score *d*.*S* is a function of its representation *d*.**F**, the expected score can be calculated by using the distribution of *d*.**F** given the confidence scores of the document (Papoulis 1984):

5 $$ E[d.S|{\bf o}]=\sum_{{\bf f} \in {d.{\bf F}}}{score({\bf f})\; P(d.{\bf F}={\bf f}|{\bf o})} $$

where *E*[*d*.*S*|**o**] is the expected score given the confidence scores **o**. Furthermore, the scores’ variance is (Papoulis 1984):

6 $$ \hbox{var}[d.S|{\bf o}] = E[d.S^2|{\bf o}]-E[d.S|{\bf o}]^2 $$

with

7 $$ E[d.S^2|{\bf o}]=\sum_{{\bf f} \in {d.{\bf F}}}{score({\bf f})^2\; P(d.{\bf F}={\bf f}|{\bf o})} $$

where *E*[*d*.*S* ^2^|**o**] is the expected squared score. Similar to the greedy algorithm in Eq. (3) of the mean-variance analysis framework, the URR framework finally ranks documents by the expected score plus a weighted expression of the scores’ standard deviation:

8 $$ RSV(d) = E[d.S|{\bf o}] - b\; \sqrt{\hbox{var}[d.S|{\bf o}]} $$

where *RSV*(*d*) is the final ranking status value by which document *d* is ranked, *E*[*d*.*S*|**o**] is the expected score of document *d* in Eq. (5), *b* represents the risk-attitude of the search engine, and $\sqrt{\hbox{var}[d.S|{\bf o}]}$ is the scores’ standard deviation in Eq. (6). Equation (8) is the general ranking framework proposed in this paper. In the following Sects. 4 and 5 we adapt the URR framework for two particular basic ranking functions for particular representations.

## Shot retrieval

In this section we describe an adaptation of the URR framework to shot retrieval in which the expected score component is equivalent to the Probabilistic Framework of Unobservable Binary (PRFUBE), which was originally proposed by Aly et al. (2008). Additional to the expected score, we define the scores’ standard deviation. For consistency reasons we use the name PRFUBE for our method for shot retrieval, despite the additional consideration of the scores’ standard deviation.

### Representation and ranking function

The PRFUBE considers binary concept-based representations, where each concept either occurs or is absent in shot. By using the analogy of concept occurrences in shots and index term assignments to documents, PRFUBE re-uses the probability of relevance given index term assignments (Robertson et al. 1981) as a ranking function:

9 $$ score({{\bf c})}=P(R|{\bf C}{=}{{\bf c})}=\frac{P({\bf C}{=}{{\bf c}|}R)\; P(R)}{P({\bf C}{=}{{\bf c})}} $$

where *P*(*R*|**C** = **c**) is the probability of relevance given that the concept occurrences **c** of the concept-based representation **C**,  *P*(**C** = **c**|*R*) is the probability of the concept occurrences **c** given relevance, *P*(**C** = **c**) is the prior of the concept occurrences **c**, and *P*(*R*) is the relevance prior. Because of the uncertainty of concept occurrences **c**, we use the ranking function in Eq. (9) as a basic ranking function in the URR framework.

### Framework integration

The integration of the ranking function in Eq. (9) into the URR framework requires the definition of a random variable for the uncertain representation and its expected score. Let *d*.**C** be the uncertain binary concept-based representation of document *d*, and let *d*.*S* = *score*(*d*.**C**) be the uncertain score of document *d* define in Eq. (9). We now define the expected score and the expected squared score which we used in the URR framework in Eq. (5) and in Eq. (7). The expected score of document *d* is:

10 $$ E[d.S|{\bf o}]=\sum_{{\bf c}\in {d.{\bf C}}}{score({{\bf c})}\; P(d.{\bf C}={\bf c}|{\bf o})} $$

where **c** is one of |*d*.**C**| = 2^*n*^ possible representations of *n* considered concepts, and **o** are the confidence scores for document *d*. Note that the calculation in Eq. (10) has a run-time complexity of *O*(2^*n*^), which makes it inapplicable to realistic numbers of concepts. We make the following independence assumptions to make the computation efficient:

11 $$ P({\bf C}|R) =\prod_i^n{P(C_i|R)} $$

12 $$ P({\bf C}) =\prod_i^n{P(C_i)} $$

13 $$ P(d.{\bf C}|{\bf o}) =\prod_i^n{P(d.C_{i}|o_i)} $$

where Eq. (11) assumes conditional independence of all random variables *C*_*i*_ given relevance, which is a common assumption in text retrieval. Following Fuhr (1989), Eq. (12) assumes that concept variables are independent in the whole collection. Finally, Eq. (13) assumes that the occurrence of concepts is independent from the occurrence of other concepts (*P*(*C*_1_, *C*_2_|*o*_1_, *o*_2_) = *P*(*C*_1_|*o*_1_, *o*_2_) (*P*(*C*_2_|*o*_1_, *o*_2_)) and from confidence scores of other concepts (*P*(*C*_1_|*o*_1_,*o*_2_) = *P*(*C*_1_|*o*_1_)). Using the above independence assumptions, the expected score in Eq. (10) can be expressed as follows:

14 $$ E[d.S|{\bf o}]=P(R)\sum_{{\bf c}\in {d.{\bf C}}}{\prod_i^n{{\frac{P(C_i=c_i|R)}{{P(C_i=c_i)}}} P(d.C_{i}=c_i|o_i)}} $$

where we can ignore the query-specific constant *P*(*R*). Additionally, because **c** is a vector of binary values, the generalized distributive law can be applied (Aji and McEliece 2000). This results in the expected score, which has a linear run-time complexity in the number of concepts:

15 $$ E[d.S|{\bf o}]= \prod_{i=1}^{n}{ \left[\underbrace{{ \frac{P(C_i|R)}{P(C_i)}}P(d.C_{i}|o_i)}_{C_i\,\hbox{occurs}} +\underbrace{{\frac{1-P(C_i|R)}{1-P(C_i)}}(1-P(d.C_{i}|o_i))}_{C_i\,\hbox{is absent}}\right]} $$

where *P*(*C*_*i*_|*R*) is the probability of concept *C*_*i*_ occurring in relevant shots, *P*(*C*_*i*_) is the prior of concept *C*_*i*_, and *o*_*i*_ is the confidence score for concept *C*_*i*_. Here, the probability *P*(*C*|*R*) is a weight which has to be defined for each query, and the prior *P*(*C*), which can be estimated from the data, see Sect. 6. Furthermore, for the calculation of the scores’ standard deviation in Eq. (6), we also require the expected squared score:

16 $$ E[d.S^2|{\bf o}] = \sum_{{{\bf c}}\in {d.{\bf C}}}{score({{\bf c})}^2 P(d.{\bf C}={{\bf c}|}{\bf o})} $$

The calculation in Eq. (16) also has a run-time complexity of *O*(2^*n*^). Using similar assumptions and derivations as in Eq. (14) and in Eq. (15), we can derive a more efficient function for the expected squared score:

17 $$ E[d.S^2|{\bf o}]= \prod_{i=1}^{n} \left[\left[\frac{P(C_i|R)}{P(C_i)}\right]^{2} P(d.C_{i}|o_i) +\left[\frac{1-P(C_i|R)}{1-P(C_i)}\right]^{2} (1-P(d.C_{i}|o_i))\right] $$

where the parameters are the same as in Eq. (15). The expected score in Eq. (15) and the standard deviation [calculated using the expected squared score in Eq. (17)] can then be used to calculate the URR retrieval score in Eq. (8).

## Segment retrieval

In this section we describe the Uncertain Concept Language Model (UCLM) ranking function for segment retrieval, which was originally presented in Aly et al. (2010). While the original publication already contained the main ideas of the URR framework, it was specific to the representation of document representations of concept frequencies and concept language model as a ranking function. In this paper, we describe the UCLM as an instance of the URR framework.

### Representation and ranking function

**Fig. 4:**
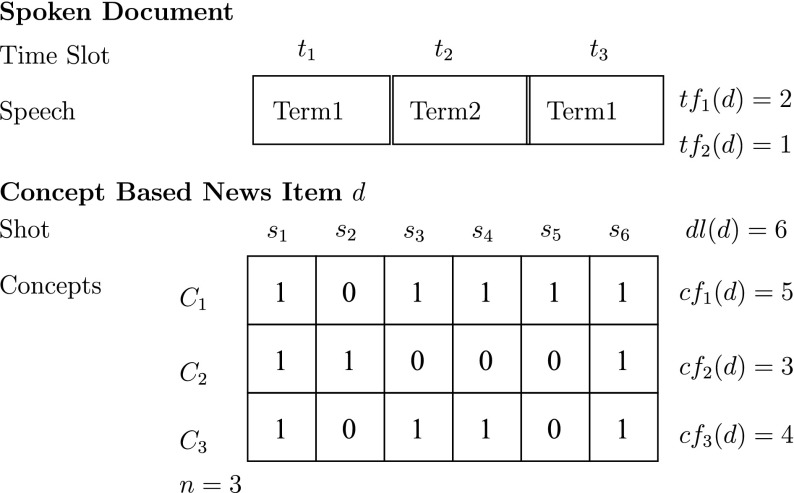
A concept-based segment representation and its analogy to a spoken document. Note that, compared to the main text, we use here the shorter notation *s*_*j*_ for shot *d*.*s*_*j*_

We model a long segment, for example a news item, as a sequence of shots. Figure 4 shows the analogy between spoken text consisting of three spoken words, and a segment consisting of the occurrence of three shots. We denote the *j*th shot of a segment *d* as *d*.*s*_*j*_, and the occurrence of a concept *C*_*i*_ in *d*.*s*_*j*_ as $C_i(d.s_j) \in \{0,1\}$. If we know the concept occurrences in each shot of a segment, we can represent a segment by its concept frequencies, in an analogy to the term frequency of a spoken text, as a sum of occurrences: *CF*_*i*_(*d*) = ∑_*j*_^*dl*^ *C*_*i*_(*d*.*s*_*j*_), where *dl* is the segment length in the number of shots. For example, the segment in Fig. 4 would be represented by the concept frequency vector **CF**(*d*) = (5, 3, 1) meaning that there are five concept occurrences of the first concept, three of the second concept, and one of the third concept.

Based on the representation of concept frequencies, we define a ranking function, which is derived from the language modeling framework (Hiemstra 2001). The basic idea behind our approach is to consider the occurrence and absence of a concept as two concept words of the language of this concept, and instead of a single stream of terms, we have multiple concept streams. We then use the language model ranking function with Dirichlet smoothing (Zhai and Lafferty 2004) as a ranking function:

18 $$ score({\bf cf}) = \prod_i^n{ {\frac{cf_i +\mu\; P(C_i|{\mathcal{D}})}{dl+\mu}} } $$

where **cf** is a vector of *n* concept frequencies, *C*_*i*_ refers to the *i*th selected concept, *cf*_*i*_ is the concept frequency of concept $C_i,\,P(C_i|\mathcal{D})$ is the prior of encountering concept *C*_*i*_,  *dl* is the segment length (in numbers of shots), and μ is the Dirichlet parameter. Note that in this setting, the segment length *dl* is always known, since we assume a perfect segmentation of videos.

### Framework integration

Because the concept occurrences in each shot are uncertain, the concept frequencies of the surrounding segment are also uncertain. Therefore, we introduce for each segment *d* a random variable for its representation consisting of concept frequencies $d.{\bf CF}=(d.CF_1,\ldots,d.CF_n)$, where *d*.*CF*_*i*_ is the uncertain concept frequency of concept *C*_*i*_. As the representation of segment *d* is uncertain, so is the concept language score in Eq. (18), for which we introduce the random variable *d*.*S* = *score*(*d*.**CF**). The expected score and the expected squared score are:

19 $$ E[d.S|{\bf o}] = \sum_{{\bf cf} \in {d.{{\bf CF}}}}{score({\bf cf}) \; P(d.{{\bf CF}}={\bf cf}|{\bf o})} $$

20 $$ E[d.S^2|{\bf o}] = \sum_{{\bf cf} \in {d.{{\bf CF}}}}{score({\bf cf})^2\; P(d.{{\bf CF}}={\bf cf}|{\bf o})} $$

where **cf** is one of |*d*.**CF**| = (*dl* + 1)^*n*^ possible concept frequency representations of *n* concepts in a segment with *dl* shots, *P*(*d*.**CF** = **cf**|**o**) is the probability that segment *d* has the concept frequencies **cf**. For example, the probability of a concept frequency one for a concept *C*_*i*_ in segment *d* with segment length *dl* = 3 is:

21 $$ P(d.CF_i=1|{\bf o}) =P(d.{\bf C}_i=(1,0,0)|{\bf o}) +P(d.{\bf C}_i=(0,1,0)|{\bf o}) +P(d.{\bf C}_i=(0,0,1)|{\bf o}) $$

where *d*.**C** is a short form for $(d.s_{1}.C,\; d.s_{2}.C,\;d.s_{3}.C)$. Because of the independence assumptions in Eq. (13), the probability of a sequence of concept occurrences **c** in a segment in Eq. (21) for concept *C* is:

$$ P(d.{\bf C}={{\bf c}|}{\bf o}) = \prod_{j=1}^{dl} { P(d.s_{j}.C=c_j|o(d.s_j)) } $$

where *o*(*d*.*s*_*j*_) is the confidence score of concept *C* in shot *d*.*s*_*j*_. Finally, the probability that a segment has the concept frequency representation **cf** can be calculated as follows:

22 $$ P(d.{{\bf CF}}={\bf cf}|{\bf o})=\prod_i^n{P(d.CF_i=cf_i|{\bf o})} $$

where *P*(*d*.*CF*_*i*_ = *cf*_*i*_|**o**) is calculated according to Eq. (21). In general, Eq. (22) can be used to calculate the expected score in Eq. (19) and expected squared score in Eq. (20) which to rank segments according to the URR ranking function in Eq. (8). However, the high number of possible representations prohibits a direct calculation of the above formulae. To reduce the computational costs, we use the Monte Carlo Sampling method (Liu 2002) to approximate the expectations in Eq. (19) and in Eq. (20): we first generate *NS* random samples of concept frequency representations, **cf** ^1^, … , **cf** ^*NS*^, from the distribution *P*(*d*.**CF**|**o**). We generate a sample of a concept frequency of concept *C*_*i*_ for segment *d* by using the concept occurrence probabilities of each shot:

$$ cf^{k}_{i} = \sum_{j=1}^{dl}\left[ (rnd() < P({d.s_{j}.C_i}|{\bf o}))\; ?\; 1\; :\; 0 \right] $$

where *k* is the index of the sample, *C*_*i*_ is the considered concept, and *rnd*() generates a uniform random number in the interval [0:1]. The notation (*X*) ? *Y* : *Z* has the following meaning: if the generated random number is lower than the probability of concept occurrence in shot *j* (*X*), we increase the concept frequency of the sample by 1 (*Y*), otherwise the frequency is left unchanged (*Z*). We repeat this procedure for all considered concepts in the representation for each of the *NS* samples. Note that the samples can be generated at indexing time to reduce computational costs at query time. The Monte Carlo estimate for the expected score in Eq. (19) and the expected squared score in Eq. (20) is then:

$$ \begin{aligned} E[d.S|{\bf o}] &\simeq {\frac{1}{NS}} \sum_{k=1}^{NS}{score({\bf cf}^k)} \\ E[d.S^2|{\bf o}] &\simeq {\frac{1}{NS}} \sum_{k=1}^{NS}{score({\bf cf}^k)^2} \end{aligned} $$

where both approximations have a linear run-time complexity in the number of samples *NS*. Because the standard error of the Monte Carlo estimate is in the order of $1/\sqrt{NS}$, a good estimate is already achieved with relatively few samples. Note that there are more advanced sampling methods which further reduce the required samples, for example importance sampling (Liu 2002). But here we focus on the qualitative results of sampling and leave more advanced sampling methods for future work.

## Experiments

In this section we present the experiments which we undertook to evaluate the performance of the URR framework. We investigated two retrieval tasks in connection with the annual TRECVid evaluation workshop (Smeaton et al. 2006): the automatic shot retrieval task, which is a standard task in TRECVid, and the segment retrieval task, which we proposed earlier to accommodate the user’s need to search for longer segments (Aly et al. 2010). Note that because we focus on purely concept-based search the performance figures presented in this section are not directly comparable with figures reported elsewhere which also use features such as text and visual similarity.

### Experiment setup

In the following we describe the general experimental setup. Table 1 shows statistics of the collections used. We used the output of state-of-the-art concept detectors which were released by participants of the TRECVid workshops. For the segment retrieval task, we used a segmentation of broadcast news videos into news items from the tv05t and tv06t collection, which was provided by Hsu et al. (2006). The segmentation resulted in 2,451 news items and 5,380 news items respectively.

**Table 1:** Statistics of the collections used in the experiments

Collection	Shots	Domain	Queries	Detectors sets	Number of concepts	Training collection for ADCS
tv05t	45,765	News	24	MM101	101	tv05d
tv06t	79,484	News	24	Vireo	374	tv05d
tv07t	18,142	G.TV	24	Vireo	374	tv05d
tv08t	35,766	G.TV	48	Vireo	374	tv05d
tv08t	35,766	G.TV	48	MM09	64	tv07d
tv09t	61,384	G.TV	24	MM09	64	tv07d

*tvXXt* TRECVid test collection of year 20XX,* News* Broadcast News,* G.TV* General Dutch Television. The detector sets are described in the following publications: MM101 (Snoek et al. 2006), Vireo (Jiang et al. 2010), MM09 (Snoek et al. 2008)

Some ranking functions use concept priors in their formula, which we estimated from the data:

$$ P(C)={\frac{\sum_{d}{P(d.C|{\bf o})}}{N}} $$

where *P*(*C*) is the concept prior of concept *C*,  *P*(*d*.*C*|**o**) is the posterior probability of concept *C* in shot *d*, and *N* is the number of shots in the collection.

Before we execute a query we first needed to select concepts and estimate the corresponding ranking function parameters. We used the Annotation-Driven Concept Selection (ADCS) which showed good performance on several collections (Aly et al. 2009). The ADCS method is based on a collection with known concept occurrences and textual shot descriptions. The probability of a concept occurrence given relevance was estimated by executing the textual query on the shot descriptions and using the known concept occurrences for the estimation of the probability (Aly et al. 2009). The shot descriptions consisted of the automatic speech recognition output together with the corresponding Wikipedia articles of the occurring concepts. We used the general-purpose retrieval engine PF/Tijah (Hiemstra et al. 2006) to rank the shot descriptions in the training collection. The parameter *m* of the ADCS method states the numbers of top-ranked shot descriptions we assume are relevant. For each concept, the method estimates the probability of the concept’s occurrence given relevance, *P*(*C*|*R*). To select concepts, we used these estimates together with the concept priors to calculate the Mutual Information between a concept and relevance which was identified by Huurnink et al. (2008) as a measure of usefulness. From the resulting ranked list of concepts, we selected the first *n* concepts.

The performance of current concept detectors is still limited, and the resulting search performance is low compared to, for example, performance figures from text retrieval. Therefore we also used our simulation-based approach (Aly et al. 2012) to investigate the search performance of the considered ranking functions with increased detector performance. This is in line with work reported in Toharia et al. (2009) which artificially varied the quality of concept detector performance in order to study the impact of improving or degrading this, on retrieval.

In the simulation the confidence scores of the positive and the negative class of known concept occurrences are modeled as Gaussian distributions. Changes in detector performance are simulated by changing the Gaussians’ parameters. For each concept in each shot we generated confidence scores randomly from the Gaussian corresponding to the concept occurrence status. On the resulting collection of confidence scores, we executed the considered ranking functions, resulting in the average precision of each method with these confidence scores. We repeated this procedure 25 times, yielding an estimation of the search performance we would expect for retrieval using detectors with these parameters. To keep our discussion focused, we only investigate the search performance when changing the confidence scores’ mean of the positive class—therefore making the detector on average more confident about the concept occurrences. For a more detailed description of this simulation approach, we refer the interested reader to Aly et al. (2012).

### Shot retrieval

In this section we describe the evaluation of our shot retrieval model PRFUBE described in Sect. 4. Table 2 shows the ranking functions to which we compared the PRFUBE. Note that it would have been interesting to compare PRFUBE with the Probabilistic Model for combining diverse Knowledge Sources in Multimedia by Yan (2006). However, we were not able to include this ranking function because it required confidence scores on a development collection which are only available for the text collection tv05t. In the following, we present the results from first investigating the influence of the risk parameter *b* on the search results, the results of using the user study for concept selection and the results from using automatic concept selection via the ADCS method.

**Table 2:** Considered ranking functions (Rank Func.) for shot retrieval (*c*′ binary detector output $(P(C|o)>0.5\rightarrow c'=1),\,p=P(C|R),\,q=P(C|\bar{R})\sim P(C)$)

Rank Func.	Description	Definition
CombMNZ	Multiply non-zero	$\prod\nolimits_{i}{P(C_i|o_i)}\;\hbox{with}\; P(C_i|o_i)>0$
CombSUM	Unweighted sum of scores	∑_*i*_ * P*(*C*_*i*_|*o*_*i*_)
PMIWS	Pointwise mutual information weighting scheme	$\sum\nolimits_i\;{\log\left({\frac{P(C_i|R)}{P(C_i)}}\right)P(C_i|o_i)}$
Borda	Rank based	∑_*i*_ * rank*(*P*(*C*_*i*_|*o*_*i*_))
BIM	Binary independence model	$\sum\nolimits_{i}\;{c'_i \log\Big({\frac{p(1-q)}{q(1-p)}}\Big)}$
ELM	Expected concept occurrence language model (λ = 0.1)	$\prod\nolimits_i\;{\left[\lambda P(C_i|o_i) + (1-\lambda) P(C_i|\mathcal{D})\right]}$

#### Risk parameter study

**Fig. 5:**
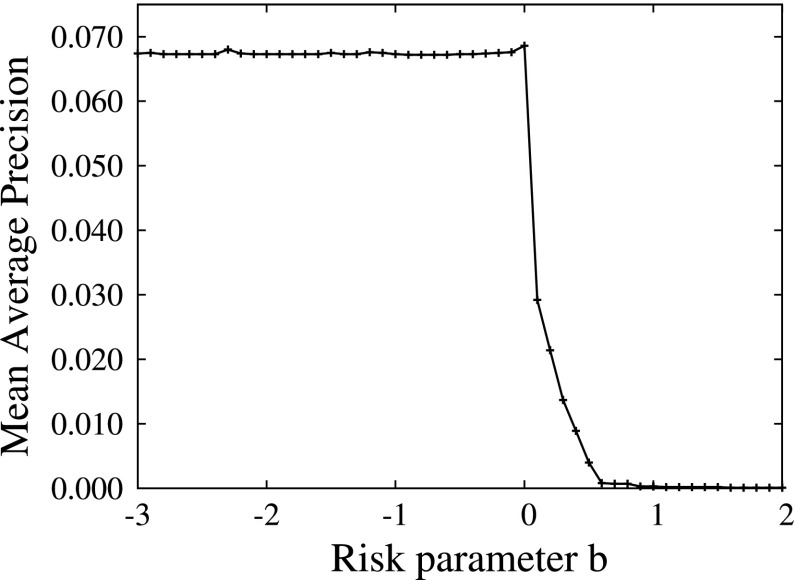
Risk parameter *b* for the ranking function $RSV(d)=E[d.S|{\bf o}]- b\;\sqrt{\hbox{var}[d.S|{\bf o}]}$

Figure 5 shows the influence of the risk parameter *b* on the search performance of PRFUBE in the tv05t collection. For a risk-averse attitude, *b* > 0, the search performance quickly decreases to virtually zero and for a risk-loving or risk-neutral attitude, *b* ≤ 0, the search performance stays approximately the same. These results were similar in the other collections investigated. Therefore, in the following we used a risk-neutral *b* = 0 attitude for PRFUBE as it provided the best performance.

#### Performance comparison

Table 3 summarizes the retrieval performance of the seven considered ranking functions over five collections with automatically selected concepts using the ADCS method. For each ranking function, the table reports three numbers. First, the optimal performance, in mean average precision (MAP), the method achieved, second, the cut-off value *m*, and finally, the number of concepts *n* used to achieve this performance. On the right, the average rank of the method over the six runs is reported. The PRFUBE is, on average, the best ranking function. In three out of six runs, PRFUBE was the best performing ranking function. In the remaining runs, its performance was the second best and not significantly worse than the best run. When taking the queries of all collections together, the MAP of the PRFUBE was significantly better than the one of the *ELM* method and the PMIWS method.

**Table 3:** Mean average precision of the ranking functions described in Table 2

Collection	tv05t	tv06t	tv07t	tv08t	tv08t	tv09t	Avg. rank
Rank Func.	MM101	Vireo	Vireo	Vireo	MM09	MM09	
CombMNZ	0.064	$0.033^{\dagger}$	0.028	$0.024^{\dagger}$	$0.042^{\dagger}$	$0.045^{\dagger}$	4.7
	10/8	700/30	100/20	10/15	100/30	100/10	
PMIWS	0.054	0.039	0.021	${\mathbf 0.041}$	**0.058**	0.067	2.7
	100/8	200/30	200/15	50/4	50/4	50/2	
Borda	$0.050^{\dagger}$	$0.012^{\dagger}$	$0.020^{\dagger}$	0.030	$0.045^{\dagger}$	0.058	5.5
	10/15	100/10	50/20	10/15	10/2	10/8	
BIM	$0.044^{\dagger}$	$0.024 ^{\dagger}$	0.026	0.037	0.050	0.063	4.8
	10/8	100/2	100/8	100/4	50/2	50/2	
ELM	**0.071**	0.040	0.031	0.040	0.050	0.064	2.3
	10/8	600/30	50/10	100/4	10/2	50/2	
PRFUBE	0.069	**0.043**	**0.039**	0.041	0.056	**0.068**	1.5
	150/10	600/30	100/45	100/4	100/4	50/2	

For each ranking function in each collection, three values are shown: first, the search performance in MAP, second, the number of document considered by the ADCS method (*m*), and finally the number of considered concepts (*n*). The $\dagger$ symbol indicates that the method is significantly *worse* than the best method for this collection, according to a two-sided, paired Wilcoxon signed rank test with a significance level of 0.05

### Segment retrieval

We now describe the experiments we undertook to evaluate the performance of the UCLM ranking function from Sect. 5 for segment retrieval. Because of the novelty of the segment retrieval task there is no standard set of queries. Therefore we decided on using the official queries for the tv05t and tv06t collections, replacing the common prefix “*Find shots of* \ldots” with “*Find news items about* \ldots” . Furthermore, we assumed that a news item is relevant to a given query if it contains at least one relevant shot, which we determined from the relevance judgments for the respective shot retrieval task. We propose that for most queries this is realistic since the user could be searching for the news item as a whole, rather than for shots within the news item.^5^

To the best of our knowledge, no comparable ranking functions exist for the segment retrieval task. Therefore, we compared the UCLM ranking function against extensions of the shot ranking functions from Table 2 and a ranking function which is similar to the one from spoken document retrieval. To use the shot ranking functions for segment retrieval, we used the average probability of concept occurrence in the shots of a segment as the normalized confidence score of the segment^6^:

$$ P(d.C|o_d) =\frac{\sum_j{P({d.s_j}.C|o_d)}}{dl} $$

where *P*(*d*.*C*|*o*_*d*_) is the normalized average occurrence probability of concept *C*. Furthermore, using similar analogies of concept occurrences and term utterances as in Sect. 5, we investigated two variants of the language modeling framework. First, we used for every concept its most likely binary state (assuming a concept occurs if *P*(*d*.*C*|*o*_*d*_) > 0.5) and determined the concept frequencies through counting. Segments were then ranked using the language modeling framework with Dirichlet smoothing (Zhai and Lafferty 2004):

23 $$ \hbox{Best-1}({\bf cf}) = \prod_i^n{{\frac{cf_i +\mu\; P(C_i|{\mathcal{D}})}{dl+\mu}} } $$

where *cf*_*i*_ is the concept frequency of concept *C*_*i*_. We refer to this ranking function as the Best-1 function. Second, we transferred the ranking function from Chia et al. (2008), which was originally proposed for spoken document retrieval, to a concept-based ranking function, referred to as the expected concept frequency language model ECFLM. The ECFLM method is based on representations of expected concept frequencies, where the expected concept frequency of a single concept is defined as:

24 $$ E[d.CF_i|{\bf o}] = \sum_{j=1}^{dl}{P(d.s_{j}.C_i|o_i(d.s_j))} $$

where *E*[*d*.*CF*_*i*_|**o**] is the expected concept frequency and *P*(*d*.*s*_*j*_.*C*_*i*_|*o*_*i*_(*d*.*s*_*j*_)) is the occurrence probability of concept *C*_*i*_ in shot *d*.*s*_*j*_. Similar to the Best-1 ranking function, the ECFLM ranks segments using the language model ranking function in Eq. (23) replacing the concept frequency *cf*_*i*_ with the expected concept frequency in Eq. (24).

To rule out random effects when generating samples for the UCLM method, see Sect. 5, we repeated each run ten times and reported the average.

#### Risk parameter study

**Fig. 6:**
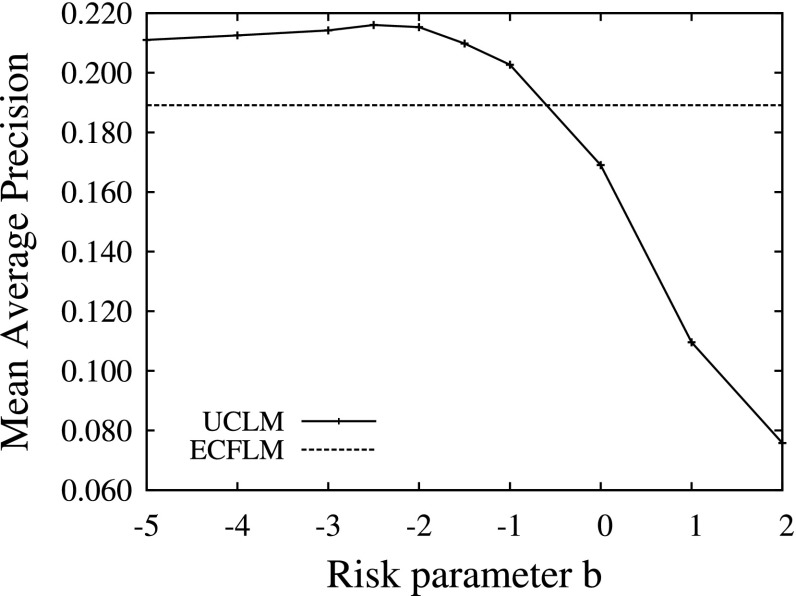
Risk parameter *b* for the ranking function $RSV(d)=E[d.S|{\bf o}]- b\;\sqrt{\hbox{var}[d.S|{\bf o}]}$

Figure 6 shows a parameter study of the UCLM ranking function on the tv05t collection. The horizontal line represents the search performance of the ECFLM ranking function which is independent of the considered risk. With a risk parameter larger than *b* > −1, the search performance of the UCLM ranking function deteriorated. For values of *b* ≥ −1 the method improved over the ECFLM method, and reached its maximum at *b* = −2. We performed similar parameters studies for the Dirichlet parameter μ and the required number of samples *NS*, see 5. In both cases, the UCLM ranking function was robust against parameter changes. We used *NS* = 200 samples, a Dirichlet parameter of μ = 60, and a risk factor *b* = −2 for the following experiments.

#### Performance comparison

Table 4 shows the comparison results of the described ranking function with the proposed UCLM ranking function. The first column for each collection indicates the number of concepts under which each ranking function performed the best. We see that the ranking functions CombMNZ, CombSUM, PMIWS, Borda, and Best-1 perform worse than the two ranking functions ECFLM and UCLM. The search performance of the UCLM ranking function is 0.214 MAP for the tv05t and 0.135 for the tv06t collection respectively. The improvement of the UCLM ranking function against all other ranking functions was significant according to a two-sided, paired Wilcoxon signed rank test with a significance level of 0.05.

**Table 4:** Results of comparing the proposed UCLM framework against four other methods described in related work

Ranking function	tv05t			tv06t		
	Concepts *n*	MAP	P10	Concepts *n*	MAP	P10
CombMNZ	10	0.105	0.045	8	0.034	0.040
PMIWS	6	0.102	0.080	2	0.050	0.065
Borda	1	0.090	0.000	2	0.052	0.061
Best-1	5	0.094	0.245	6	0.073	0.083
ECFLM	10	0.192	0.287	32	0.101	0.143
UCLM	10	0.214^*^	0.291	18	0.135^*^	0.151

The * symbol indicates that the improvement of the UCLM framework compared to this ranking function were significant according to a two-sided, paired Wilcoxon signed rank test with a significance level of 0.05 against all other methods

### Simulated concept detectors

**Fig. 7:**
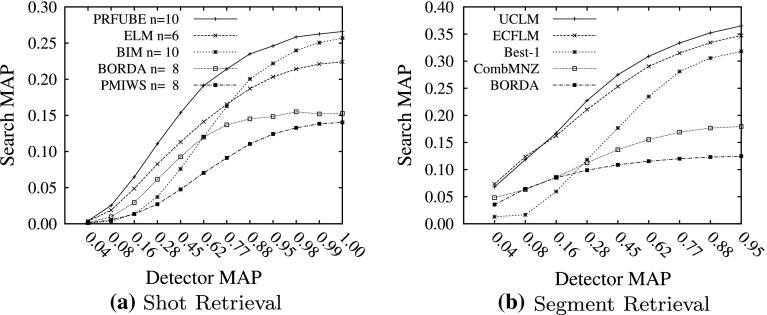
Results from simulated concept detectors changing the mean of the positive class μ_1_ using realistically set parameters

In this section we describe the results we obtained by simulating the outputs of concept detectors. The simulation procedure required a collection with known concept occurrences, for which we used the *tv*05*d* collection. To make the concept selection realistic, we divided the collection into a test and development set (mm.dev and mm.test respectively) according to Snoek et al. (2006)^7^. Figure 7 shows the results of improved detector performance on improved search performance for the mm.dev collection with realistically set weights estimated by ADCS^8^. The x-axis shows the increase in detector performance in terms of MAP which resulted from the increase of the mean confidence scores of the shots in which the concept occurs. The y-axis shows the resulting expected search performance in terms of MAP. Figure 7a shows that the PRFUBE method consistently performs better than the other ranking functions at all levels of concept detector performance. With high detector performance, the search performance of the PRUFBE ranking function and the BIM ranking function converges, as both rankings are similar under perfect detection.

Figure 7b shows the simulation results for the segment retrieval task. At low detector performance, the UCLM ranking function performs practically identical to the ECFLM ranking function. With a higher detector performance, the UCLM ranking function wins in performance. The Best-1 ranking function increases performance only with much higher detector performance.

### Influence of the scores’ standard deviation

**Fig. 8:**
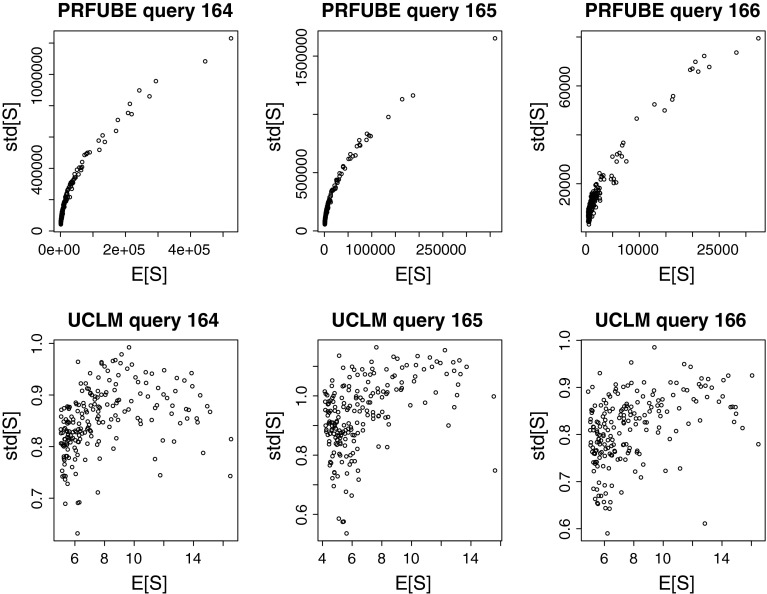
The relationship between expected score and standard deviation of the PRFUBE method and the UCLM method on the tv05t collection

For the PRFUBE, the consideration of the scores’ standard deviation did not improve performance, see Fig. 5, while it did for the UCLM method, see Fig. 6. Therefore, we investigated whether the reason for this lies in the relationship between the expected scores and the scores’ standard deviation of the respective function. Note that for a risk-loving attitude (*b* > 0), if the standard deviation $\sqrt{\hbox{var}[d.S]}$ increases monotonically with the expected score *E*[*d*.*S*], it does not affect the ranking compared to only using the expected score. Figure 8 plots the expected score *E*[*d*.*S*] (x-axis) against the standard deviation $\sqrt{\hbox{var}[d.S]}$ (y-axis) for the 200 highest ranked documents of the given queries in the tv05t collection. For PRFUBE, the standard deviation is roughly monotonically increasing with the expected score, while for UCLM there is much more variability. The results for other queries and collections were similar.

## Discussion

We now discuss the experimental results obtained in the previous section.

### Effectiveness

Both derivations of the URR framework, PRFUBE and UCLM, showed significant improvement over most other retrieval methods from other uncertainty classes, as shown in Tables 3 and 4. Furthermore, according to the simulations presented in Fig. 7, both methods will also continue having a strong performance compared to other methods as concept detector performance improves.

### Robustness

Given the relative low overall performance numbers, strong performance in some collections could be caused by particular “lucky” detections in relevant shots. Therefore, a robust retrieval method is not only effective (has good performance in many collections) but also stable (performs similar across collections). Table 3 shows that the PRFUBE is robust in six different collections. Similarly, the UCLM method performed stably for two collections. Furthermore, the detector simulation experiments in Fig. 7 suggest that the performance improvements are robust against changes of detectors.

### Risk-attitude

In both instances of the URR framework, a risk-neutral or risk-loving attitude helped performance. For the PRFUBE, the risk-loving attitude did not increase performance. We propose that the almost monotonic relationship between expected score and standard deviation in Fig. 8 is the reason why the standard deviation does not improve the ranking for PRFUBE. We expect that the practically monotonic relationship of expected score and standard deviation of the PRFUBE originates from the independence assumptions made in Eq. (11)–(13), which are known not to match the data (Cooper 1995), and propose further investigations for future work. For the UCLM, there was much higher variability in the standard deviation compared to the expected scores, giving the standard deviation the possibility to improve the ranking. Here, a risk-loving attitude improved performance significantly over the strongest baseline.

## Conclusions

In summary, we proposed the URR framework that meets the challenge to define *effective* and *robust* ranking functions in concept-based video retrieval under detector uncertainty. While the framework is independent of the retrieval task, we adapted it to the tasks of retrieving *shots* and *(long) segments*. For shot retrieval, our framework improved over five baselines on six collections, and for segment retrieval, it improved significantly over four baselines on two collections. Furthermore, when simulating improved concept detectors these improvements prevailed. We now discuss our conclusions in more detail.

The URR framework considers basic ranking functions adapted from text retrieval based on representations of known concept occurrences. The uncertainty of detectors is handled separately: the framework takes into account multiple concept-based representations per document. It uses the confidence scores of detectors to assign each representation a probability of being the correct representation. The application of the considered basic ranking function to the multiple representations results in multiple scores for each document. Inspired by the mean-variance analysis framework by Wang (2009), the URR framework ranks documents by the expected score plus a weighted expression of the scores’ standard deviation, which represents the chance that scores are actually higher than the expected score. We demonstrated the ability of the general framework to produce effective and robust ranking functions by applying it to two retrieval tasks: shot retrieval and segment retrieval.

For shot retrieval, the framework used the probability of relevance given concept occurrences as a ranking function, which was derived from the probability of relevance ranking function originally proposed in text retrieval (Robertson et al. 1981). In terms of mean average precision, this ranking function improved over six baselines, representing other approaches to detector uncertainty, on three out of six collections. For the collections where it showed poorer performance than others, those were not significant. When considering all queries of the six collections together, the improvements over all baselines were significant. For segment retrieval, we proposed that ranking functions should include the *within-segment importance* when retrieving long segments. We used the concept frequency to represent the within-segment importance. We calculated the expected score and scores’ standard deviation by Monte Carlo Sampling to reduce prohibitively large number of possible representations, using 200 samples. Based on the representation of concept frequencies we used the concept language model as a ranking function, which was originally proposed in Aly et al. (2010) and derived from language models in text retrieval, see Hiemstra (2001). We showed through simulation experiments that the search performance improves with improved detectors. Based on these results, we conclude that the application of the URR framework results in effective ranking functions.

For ranking functions to be *robust*, the URR framework explicitly modeled the risk-neutral choice and the risk of choosing this score by the expected score and the scores’ standard deviation respectively. We found that a risk-averse attitude resulted in poor performance for both retrieval tasks. For shot retrieval, the consideration of the scores’ standard deviation did not improve over the condition in which only the expected score was used. 9 We found that the scores’ standard deviation often increased monotonically with the expected score, which prevents the standard deviation to influence the ranking. We attributed this behavior to the common independence assumptions made in IR, which are also made in the shot ranking function but often do not match the data (Cooper 1995). For the segment retrieval task, the use of the scores’ standard deviation significantly improved the search performance compared to the condition of exclusively using the expected score. For both retrieval tasks, the ranking functions derived from the URR framework performed between the best two systems over all considered collections and detectors. Based on these findings we conclude that the ranking functions derived from the URR framework also perform robust.

The URR framework makes few assumptions about the uncertain representation, which was done for the specific shot retrieval task and the segment retrieval task. As future work we therefore aim to apply the URR framework to other uncertain representations, for example the uncertain variants of spoken text generated by probabilistic automatic speech recognition, or the uncertain references to known entities in text retrieval. Finally, the URR framework does not consider the overall performance of concept detectors which recently received research interest (Yang and Hauptmann 2008). Therefore, we propose to extend the URR framework by measures which incorporate the overall detector performance.

## Footnotes

1 We use the terms document and video shot or a longer video segment interchangeably as both refer to retrievable units of information.

2 We use similar notation to the unusual notation *d*_*j*_.*Win* throughout this paper to prevent an excessive amount of subscripts.

3 The URR framework was originally proposed in the PhD thesis of the first author (Aly 2010).

4 Note that the distribution of *d*.**C** is discrete, although the score might be real-valued. The reason is that the arguments to *score*,  *d*.**C**, are discrete.

5 A similar assumption is made during the creation of relevance judgments for the text retrieval workshop TREC, where a document is relevant if a part of it is relevant.

6 We also investigated the use of the minimum or maximum confidence score but did not find any improvements.

7 Note that we used the development collection from Snoek et al. (2006) as a test collection since it contained more shots; making the simulation results more realistic.

8 For shot retrieval, we left out the CombMNZ ranking function since it has similar results to the PMIWS method. For segment retrieval, we left out the PMIWS since it performed similar to CombMNZ.

9 Note that the expected score is equivalent to ranking a marginalization approach which we originally proposed in Aly et al. (2008).
